# Changes in Vision-Related Quality of Life before and after Geographic Atrophy Development in Age-Related Eye Disease Study Participants

**DOI:** 10.1016/j.xops.2025.101022

**Published:** 2025-11-25

**Authors:** Minali Prasad, Susan Vitale, Elvira Agrón, Thilaka Arunachalam, Emily Y. Chew

**Affiliations:** National Eye Institute, Bethesda, Maryland

**Keywords:** Geographic atrophy, Rasch-calibration, NEI-VFQ, Quality of life

## Abstract

**Objective:**

To examine for change in vision-related quality of life before and after geographic atrophy (GA) development.

**Design:**

A post hoc analysis of a prospective randomized clinical trial.

**Participants:**

Age-Related Eye Disease Study (AREDS) participants with ≥2 study visits 1 year apart at which they completed the National Eye Institute Visual Function Questionnaire-25 (NEI VFQ-25) and age-related macular degeneration (AMD) severity gradings available at the VFQ visits.

**Methods:**

A masked reading center assessed AMD severity using annual color fundus photographs. Regression spline models with random effects for time and eye-within-participant (SAS 9.4) were used to compare the rate of change in quality of life (difference in slope for each of the 4 quality of life measures) before and after development of GA (with separate models for GA subtypes: central GA [CGA], noncentral GA [NCGA], and any GA). Models were adjusted for age and visual acuity. If neovascular AMD developed after a GA outcome, we censored the subsequent observations.

**Main Outcome Measures:**

Outcomes included the VFQ composite score calculated as an average score of nonmissing answered items. The method of successive dichotomizations was used to estimate person measures for the overall NEI VFQ-25 and for 2 derived subscales: visual functioning and social-emotional functioning.

**Results:**

Among AREDS participants with NEI VFQ-25 data available, 358 eyes (298 participants) developed any GA. None of the quality of life measures differed significantly pre- and post-CGA. Rasch-calibrated subscale score for visual function and composite scores declined more quickly after NCGA (difference in slope [post minus pre]: –0.10 logit/yr [95% confidence interval: –0.18, –0.01], *P* = 0.03; –0.78 points/yr [95% confidence interval: –1.47, –0.08], *P* = 0.03, respectively) and after any GA (–0.09 logit/yr [95% confidence interval: –0.16, –0.01], *P* = 0.02; –0.68 points/yr [95% confidence interval: –1.29, –0.07], *P* = 0.03, respectively).

**Conclusions:**

We observed worsening quality of life after development of NCGA and any GA in AREDS participants across several quality of life measures. Development of CGA was not associated with any significant changes in the quality of life measures, possibly due to the smaller sample size and limited power. Our findings highlight the importance of examining the relationship between GA subtypes and different indices of quality of life.

**Financial Disclosure(s):**

Proprietary or commercial disclosure may be found in the Footnotes and Disclosures at the end of this article.

Geographic atrophy (GA) is a form of advanced age-related macular degeneration (AMD) characterized by a focal area of hypo- or depigmentation in an area of degenerated retinal pigment epithelium without previous development of neovascular AMD (nAMD).^1^ The prevalence of GA in the United States is approximately 1 million, with 160 000 new cases diagnosed per year.^2^ Risk factors include older age and positive family history.^2^ The prevalence of unilateral GA is 1 in 5 patients among those ≥85 years of age.^3^ Patients with GA experience visual impairment affecting activities of daily life, and those with GA involving the fovea can have rapid vision loss.^2^ The median time from GA without to GA with subfoveal involvement has been estimated to be between 1.4 and 2.5 years.^2^

The impact of GA and the effectiveness of vision rehabilitation or therapeutics are often evaluated through vision-related quality of life (VRQOL) measures in addition to clinical and imaging outcomes. Patients with GA were found to have lower VRQOL scores for near and distance activities, dependency, driving, social functioning, mental health, role difficulties, color vision, and peripheral vision.^4^

There are few studies evaluating the change in vision- or health-related quality of life after GA development. Ahluwalia et al reported that the rate of VRQOL decrease was similar pre- and postdevelopment of central GA (CGA) based on the Age-Related Eye Disease Study (AREDS).^5^ To our knowledge, this is the only study evaluating the rate of change in VRQOL before and after the development of GA. They also reported that the rate of VRQOL decline was significantly greater after progression to nAMD, and that participants with CGA had a greater rate of VRQOL decline than those with nAMD. The objective of the current study was to examine changes in VRQOL (with separate analyses for visual and socioemotional functioning as well as CGA, noncentral GA [NCGA], and any GA) before and after the development of GA among AREDS participants.

## Methods

This is a secondary analysis of data collected prospectively in a randomized clinical trial. The AREDS was approved by the National Institutes of Health Institutional Review Board and adheres to the tenets of the Declaration of Helsinki. All AREDS participants provided written informed consent.

### Study Population

We used data from the AREDS (1992–2001),^6^ a randomized clinical trial designed to evaluate the effect of nutritional supplements on the risk of developing late AMD. We included participants in AREDS with ≥2 study visits at which they completed the National Eye Institute Visual Function Questionnaire-25 (NEI VFQ-25)^7^ plus a 14-item appendix regarding VRQOL (1996 version) (administered in person by trained personnel annually from December 16, 1997, to April 4, 2001)^8^ and who had information about their AMD status available at the VFQ visits. The assessment of AMD was conducted by trained graders at the University of Wisconsin Fundus Photograph Reading Center, who evaluated color fundus photographs using a standardized protocol. Geographic atrophy was defined as a sharply demarcated, usually circular zone of partial or complete depigmentation of the retinal pigment epithelium, typically with exposure of underlying large choroidal blood vessels, of at least one-eighth of disk diameter in diameter.^9^ In the subgroup of visits at which eyes had GA, the Reading Center performed additional grading of GA characteristics (noncircularity index, area of GA, and proximity to fovea).

### Clinical Outcomes

We evaluated the rate of change in VRQOL before and after the development of CGA, NCGA, and any GA. Representative images of CGA and NCGA are shown in Figure S1 (available at www.ophthalmologyscience.org). If neovascularization subsequently developed in an eye that had already developed GA, we censored all information after that time, given that the effects of neovascularization can affect the appearance of atrophic retina^10^ and could confound the relation of GA to VRQOL.

### Assessment of VRQOL

We used 2 approaches to describe VRQOL, based on AREDS participants' responses to the NEI-VFQ-25. The first was a conventional approach, computing the composite score for all items of the NEI-VFQ-25 as described previously and scored as^11^ 0 to 100, with lower scores representing worse VRQOL. Clinically significant differences can be defined as a change in the overall NEI-VFQ score or subscale score by ≥10 points.^12^ The second approach was to compute Rasch-calibrated person measures by using the method of successive dichotomizations (R package “msd”)^13^ as applied by Goldstein et al.^14^^,^^15^ Rasch analysis is a modern psychometric approach that can be used to address the scaling limitations of the original scoring methods for the NEI VFQ-25.^16^ The original scoring methods for the NEI VFQ-25 consist of taking a weighted average of the ordinal responses to the individual items, which does not create an interval-level measurement. In contrast, Rasch-based methods are used to transform the ordinal responses into interval-level person measures, producing calibrated scores that are more appropriate for statistical analyses and interpretation of change over time. The method of successive dichotomization yields 3 separate person measures: an overall measure (Rasch-calibrated overall score [M2C]) of all 25 items, a subscale describing visual function (Rasch-calibrated subscale score for visual function [M2VF]), and a subscale describing socioemotional function (Rasch-calibrated subscale describing socioemotional function [M2SE]). For the M2C, M2VF, and M2SE measures, the units are logits, and a lower score represents lower VRQOL. Clinically significant differences in the Rasch-calibrated composite score are defined as a change in M2C by 0.78 logits.^14^

### Statistical Analysis

Statistical analysis was conducted by using SAS v. 9.4 (SAS Inc). Primary analyses consisted of evaluating the change in slope of each VRQOL measure (composite score, M2C, M2VF, and M2SE) before and after developing GA (analyzing each GA outcome separately, as discussed in Clinical Outcomes) by using a regression spline model with random effects for time and eye-within-participant (SAS 9.4 Proc Mixed) to estimate slopes pre- and post-GA development and to test for differences between pre- and postslopes, adjusting for age, visual acuity (VA), and GA status in the fellow eye. We also analyzed the association between GA characteristics (GA area, proximity to fovea, and noncircularity index) and VRQOL measures; these analyses used only post-GA visit information, as this was the only time GA characteristics could be graded. We also used mediation analysis^17^ (SAS 9.4 Proc Mixed) to describe VRQOL changes post-GA development, assessing the mediating effect of VA on the effect of GA characteristics on VRQOL.

## Results

Among AREDS participants with NEI VFQ-25 data available, 358 eyes (25.3%) (298 participants) developed any GA. This represents 25.3% of 1416 eyes (of 1040 participants) with incident GA, including 382 eyes with CGA and 1034 eyes with NCGA. The average age was 74.1 years (range 59–85 years), and 54.1% were female (Table S1, available at www.ophthalmologyscience.org). Average VA in the better-seeing eye was 76.4 letters (range, 8–97 letters; Snellen equivalent 20/30, range 20/700–20/12.5). Ninety-eight eyes of 90 people developed CGA, and 260 eyes of 226 people developed NCGA.

### Changes in VRQOL before and after Development of GA

**Figure 1:**
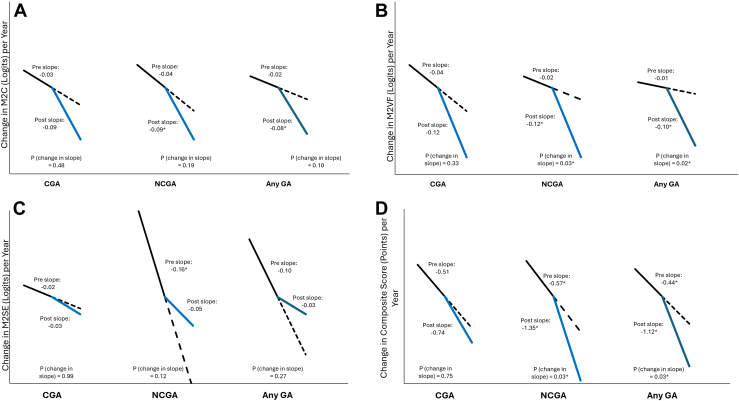
Change in M2C (Rasch-calibrated overall score) **(A)**, M2VF (Rasch-calibrated subscale score for visual function) **(B)**, M2SE (Rasch-calibrated subscale score for socioemotional function) **(C)**, and composite score **(D)** before (pre) and after (post) the development of CGA, NCGA, and any GA. CGA = central geographic atrophy; GA = geographic atrophy; NCGA = noncentral geographic atrophy.

Table S2 (available at www.ophthalmologyscience.org) and Figure 1 show the slopes pre- and postdevelopment of GA for each VRQOL outcome. All pre-GA slopes of VRQOL measures over time were negative but were only statistically significant for M2SE and the composite score for NCGA and any GA outcomes. For every 1-year change in time, there was a decrease in M2SE by 0.16 logits (95% confidence interval: –0.25 to –0.07, *P* = 0.001) and a decrease in the composite score by 0.57 points (–1.03 to –0.11, *P* = 0.02) before the development of NCGA. For every 1-year change in time, there was a decrease in M2SE by 0.10 logits (–0.17 to –0.03, *P* = 0.007) and in composite score by 0.44 points (–0.85 to –0.03, *P* = 0.03) before the development of any GA.

All post-GA slopes of VRQOL over time were negative but statistically significant only for M2C, M2VF, and the composite score for outcomes of NCGA and any GA. For every 1-year change in time, there was a decrease in M2C by 0.09 logits (–0.15 to –0.03, *P* = 0.005), a decrease in M2VF by 0.12 logits (–0.19 to –0.04, *P* = 0.002), and a decrease in composite score by 1.35 points (–1.94 to –0.76, *P* < 0.001) after the development of NCGA. For every 1-year change in time, there was a decrease in M2C by 0.08 logits (–0.13 to –0.02, *P* = 0.006), a decrease in M2VF by 0.10 (–0.16 to –0.04, *P* = 0.002), and a decrease in the composite score by 1.12 points (–1.65 to –0.59, *P* < 0.001) after the development of any GA.

For the outcome of CGA, slopes were negative for all VRQOL measures pre- and post-CGA but were not statistically significant.

When comparing the change in slope from post-GA to pre-GA, the differences were all negative, indicating that VRQOL declined more quickly after GA developed (except for M2SE), and were statistically significant for M2VF and composite score for outcomes NCGA and any GA. For M2VF, scores declined more quickly after NCGA (difference in slope [post minus pre]: –0.10 logit/yr [–0.18 to –0.01], *P* = 0.03) and after any GA (–0.09 logit/yr [–0.16 to –0.01], *P* = 0.02). Composite scores declined more quickly after NCGA (difference in slope [post minus pre]: –0.78 points/yr [–1.47 to –0.08], *P* = 0.03) and after any GA (–0.68 points/yr [–1.29, –0.07], *P* = 0.03).

### Relation to GA Characteristics

Table 1 displays the association of GA characteristics with VRQOL measures adjusted for age, time, and VA based on visits after the development of GA. Proximity of GA to the fovea and noncircularity index were not significantly associated with any of the VRQOL measures. Larger areas of GA were significantly associated with declines in each of the VRQOL measures except M2SE. For every 1 mm^2^ increase in GA area, the M2C score decreased by 0.04 logits (95% confidence interval: –0.07 to –0.02, *P* = 0.004), the M2VF score decreased by 0.05 logits (–0.07 to –0.02, *P* < 0.001), and the composite score decreased by 0.49 points (–0.75 to –0.22, *P* = 0.003).

**Table 1:** Association of GA Characteristics (Area, Proximity to Fovea, and Noncircularity Index) with VRQOL Measures Adjusted for Age, Time, and VA

	N	Mean ± SD	M2C		M2VF		M2SE		Composite Score	
			Slope [95% CI]	*P* Value	Slope [95% CI]	*P* Value	Slope [95% CI]	*P* Value	Slope [95% CI]	*P* Value
GA area (mm^2^)	236	5.29 ± 6.31	–0.04 [–0.07, –0.02]	0.004	–0.05 [–0.07, –0.02]	<0.001	–0.02 [–0.05, 0.004]	0.09	0.49 [–0.75, –0.22]	0.003
Proximity to fovea (mm)	236	220.24 ± 341.31	0.0003 [–0.0002, 0.0007]	0.22	0.0001 [–0.0004, 0.0006]	0.62	–0.0001 [–0.0006, 0.0004]	0.70	0.003 [–0.002, 0.007]	0.24
Noncircularity index	190	0.57 ± 0.24	0.29 [–0.40, 0.98]	0.54	0.34 [–0.37, 1.06]	0.50	0.18 [–0.54, 0.91]	0.62	4.05 [–2.88, 10.97]	0.25

CI = confidence interval; GA = geographic atrophy; M2C = Rasch-calibrated overall score; M2SE = Rasch-calibrated subscale describing socioemotional function; M2VF = Rasch-calibrated subscale score describing visual function; SD = standard deviation; VA = visual acuity; VRQOL = vision-related quality of life.

This analysis had reduced sample sizes because GA area, proximity to fovea, and noncircularity index were based on fundus photography, which was not completed among all participants developing GA. Additionally, graders were not able to measure noncircularity index in eyes with patchy/scattered GA.

### Mediation Analyses

**Figure 2:**
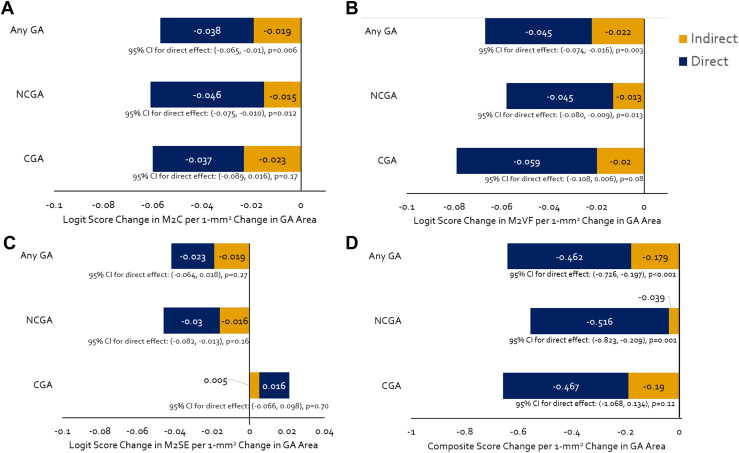
Direct effect and indirect effect (the amount of the effect that is mitigated by VA) of GA area on VRQOL measures including M2C **(A)**, M2VF **(B)**, M2SE **(C)**, and composite score **(D)**. CGA = central geographic atrophy; CI = confidence interval; GA = geographic atrophy; M2C = Rasch-calibrated overall score; M2SE = Rasch-calibrated subscale score for socioemotional function; M2VF = Rasch-calibrated subscale score describing visual function; NCGA = noncentral geographic atrophy; VRQOL = vision-related quality of life; VA = visual acuity.

We further examined the relation of GA area to VRQOL by performing a mediation analysis to assess whether the association of GA area with VRQOL could be accounted for by adjusting for the (known) effect of VA on VRQOL. Mediation analysis provides estimates for the direct effect of GA area on VRQOL and for the indirect effect (the amount of the effect that is mitigated by VA) of GA area on VRQOL. We ran separate mediation analyses for the 4 VRQOL measures and the 3 GA outcomes (Fig 2 and Tables S3–S5, available at www.ophthalmologyscience.org). For M2C, M2VF, and composite score, the direct effect of GA area on VRQOL was statistically significant after accounting for the mediating effect of VA; for NCGA and any GA, CGA had similar effect sizes but did not reach statistical significance. For every 1 mm^2^ increase in NCGA area, the M2C score decreased by 0.05 logits (–0.08 to –0.01, *P* = 0.012), M2VF score decreased by 0.05 logits (–0.08 to –0.01, *P* = 0.013), and the composite score decreased by 0.52 points (–0.82 to –0.21, *P* = 0.001) after accounting for the mediating effects of VA. For every 1 mm^2^ increase in any GA area, the M2C score decreased by 0.04 logits (–0.07 to –0.01, *P* = 0.006), the M2VF score decreased by 0.05 logits (–0.07 to –0.02, *P* = 0.003), and the composite score decreased by 0.46 points (–0.73 to –0.20, *P* < 0.001). The observed patterns differed substantially for M2SE for all GA outcomes, and none of the estimated effects were statistically significant.

## Discussion

In this analysis of AREDS, we found that visual but not socioemotional functioning declined at a faster rate after the development of NCGA and any GA. Furthermore, the area of GA was significantly associated with a decline in M2C, traditional composite score, and visual functioning. Our use of Rasch-calibrated VRQOL scores allowed for separate analysis of visual function and socioemotional functioning.

Prior studies have used patient-reported outcome measures, such as the NEI-VFQ, to investigate the impact of GA on VRQOL. For instance, lesion size, VA, and comorbidities such as chronic obstructive pulmonary disease were found to affect VRQOL assessed with the VFQ.^18^ The AREDS report no. 10 also found that the general health, general vision, and social functioning NEI-VFQ subscale scores were significantly lower among participants with advanced AMD (including GA) compared with participants with few, if any, drusen.^8^ The subscale scores appeared to linearly decrease as AMD severity increased, demonstrating the progressive worsening of VRQOL with advancing disease severity.^8^ However, there is a relative paucity of research on the rate of decline of VRQOL after the development of GA.

Ahluwalia et al similarly evaluated the progression of VRQOL after the development of CGA and nAMD using the NEI-VFQ in the AREDS cohort. The initial AREDS dataset used CGA as the main outcome. Ahluwalia et al also evaluated the study using the former outcome of either CGA development or nAMD. The current study focused on GA and added those participants who developed any GA, NCGA, which became the more updated outcome measurement. While the Ahluwalia et al report was also based on the AREDS cohort, it was limited by its treatment of pre- and post-GA slopes as independent quantities (not accounting for the correlation of pre- and post-VRQOL measurements in the same individual), confounders such as the use of vision rehabilitation services or low vision aids, and nonlinear change in VRQOL.^5^ They reported no significant difference in the rate of VRQOL decline after the development of CGA,^5^ which aligns with our findings of no significant difference in the rate of decline of M2C, M2VF, M2SE, or composite score. Given that both NCGA and any GA demonstrated a significant difference in the rate of decline of M2VF and composite score after the development of GA, it is likely that the CGA findings are due to a low sample size (n = 98 eyes in our study and n = 107 eyes in Ahluwalia et al). Considering that patients with CGA are likely to have worse VA than patients with NCGA,^19^^,^^20^ future studies with a larger sample size may demonstrate a significant difference in the rate of decline of VRQOL among patients with CGA. However, Ahluwalia et al also hypothesize that patients may adapt to the changes in their vision from the gradual progression of CGA, which may explain the similar rate of decline in VRQOL before and after the development of CGA.

We also found that the area of GA, but not proximity to fovea or noncircularity index, was associated with the rate of decline of VRQOL scores. This is congruent with prior studies that also reported an association between GA area and worse VRQOL scores^18^^,^^20^^,^^21^ and can be explained by the natural progression and pathophysiology of GA, which involves a gradually enlarging area of atrophic retinal pigment epithelium over time.^1^ While Ahluwalia et al did not find a significant association between total GA area and VRQOL, they reported that VRQOL scores were associated with area of atrophy when examining ETDRS grid subfields individually and grouped by quadrants, inner and outer rings, and vertical or horizontal halves.^22^ Other factors associated with VRQOL in GA include the best-corrected VA of the better eye, low-luminance VA for the better and worse eye, and foveal sparing status.^18^^,^^20^^,^^21^ To our knowledge, no other studies have evaluated the association between decline of VRQOL and GA characteristics, including proximity to fovea and noncircularity index.

Our mediation analyses demonstrated that after accounting for VA, the M2C, traditional composite score, and visual functioning score (M2VF) remained significantly associated with the area of NCGA and any GA. The lack of significant associations with CGA was likely due to the small sample size of eyes. The results demonstrate that the larger areas of GA are independently associated with worse VRQOL. These findings are also similar to Künzel et al,^20^^,^^21^ who reported a significant association between GA area and worse VRQOL after adjusting for VA. In contrast, Ahluwalia et al^22^ found no significant association between VRQOL and total GA area in the better or worse eye, but they did not adjust their analysis for VA.

The strengths of this study lie in the analysis of Rasch-calibrated VRQOL scores, in addition to the traditional composite score of the NEI-VFQ. The Rasch-calibrated scores allowed us to analyze VRQOL based on visual functioning and socioemotional functioning separately, using a calibrated, equal interval scale. While Ahluwalia et al similarly analyzed the change in rate of VRQOL after the development of CGA, their analysis was limited by not accounting for the correlation of pre- and post-VRQOL measurements within each participant. Additionally, we evaluated CGA and NCGA separately because of the difference in severity of visual functioning between these 2 types of atrophic AMD. Despite these strengths, the present work had limitations. Geographic atrophy development was based on color fundus photography only without examination of fundus autofluorescence images or OCT, which show signs of GA at an earlier point than fundus photography. Further, our sample size was reduced by requiring ≥2 visits 1 year apart with NEI-VFQ administrations. Additionally, there is inherent difficulty and subjectivity in interpreting VRQOL scores.

In conclusion, we found that visual functioning, but not socioemotional functioning, declined at a greater rate after the development of NCGA and any GA, and that the area of GA was significantly associated with the rate of VRQOL decline even after accounting for the mediating effects of VA. Our findings highlight the importance of examining the relationship between GA subtypes and different indices of quality of life. These results may have implications for patient counseling regarding the impact of the development of GA on activities of daily living that require visual input. Future studies could investigate factors that may underlie the difference in results between CGA and NCGA reported in our study.
