# Supplementary material for: Changes in Vision-Related Quality of Life before and after Geographic Atrophy Development in Age-Related Eye Disease Study Participants
Source: Ophthalmol Sci. 2025 Nov 25;6(2):101022. doi: 10.1016/j.xops.2025.101022 (PMC12803917; doi:10.1016/j.xops.2025.101022)
Supplement: Figure S1 [file mmc1.pdf]

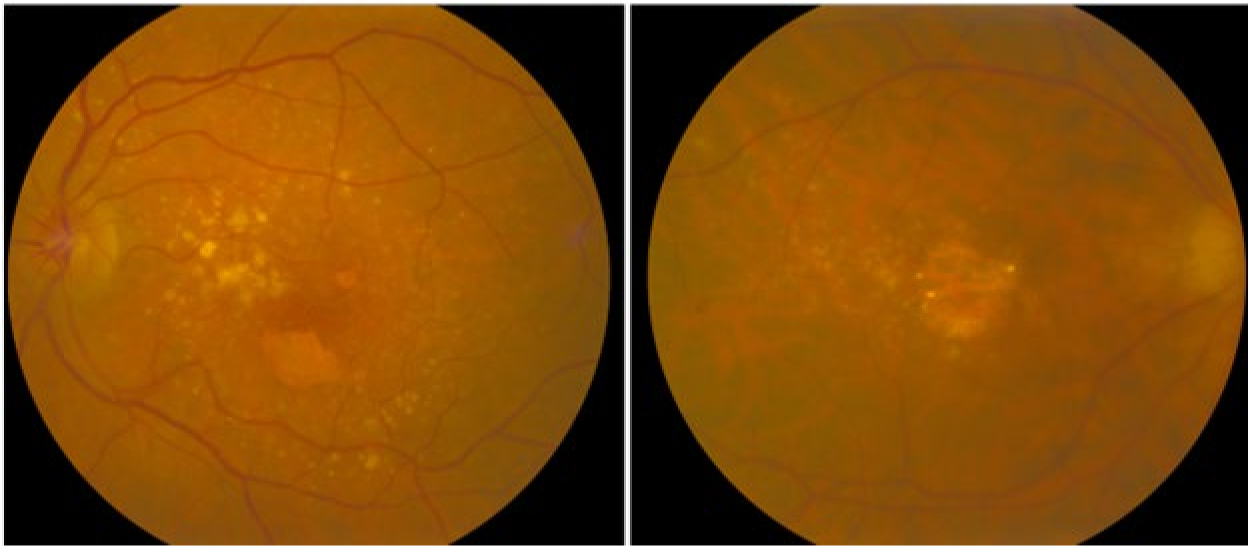

Supplementary Figure 1. Fundus photography of non-central geographic atrophy (left) and central geographic atrophy (right).
