# Supplementary material for: Changes in Vision-Related Quality of Life before and after Geographic Atrophy Development in Age-Related Eye Disease Study Participants
Source: Ophthalmol Sci. 2025 Nov 25;6(2):101022. doi: 10.1016/j.xops.2025.101022 (PMC12803917; doi:10.1016/j.xops.2025.101022)
Supplement: Table S1 [file mmc2.pdf]

Supplementary Table 1. Eye and demographic characteristics at baseline (when the NEI VFQ-25 was first administered)

	N (participants)	Mean ± Standard Deviation	Range
Age (years)	298	74.10 ± 5.41	59-85
Female	165 (54.1%)		
Race			
Non-Hispanic White	296 (99.3%)		
Non-Hispanic Black	1 (0.3%)		
Other	1 (0.3%)		
M2C	295	2.44 ± 1.36	-1.99-7
M2VF	295	2.38 ± 1.41	-2.67-7
M2SE	253	1.70 ± 1.40	-2.89-7
AVTOT	298	83.15 ± 13.08	27.53-100
Visual Acuity (letters)	354[a]	76.43 ± 12.18	8-97

Abbreviations: AVTOT, composite score for all items of the NEI VFQ-25; M2C, Rasch-calibrated overall score; M2VF, subscale score describing visual function; M2SE, subscale describing socioemotional function; NEI VFQ-25, National Eye Institute 25-item Visual Function Questionnaire.

[a]Number of eyes with a visual acuity measurement at baseline
