# Supplementary material for: Changes in Vision-Related Quality of Life before and after Geographic Atrophy Development in Age-Related Eye Disease Study Participants
Source: Ophthalmol Sci. 2025 Nov 25;6(2):101022. doi: 10.1016/j.xops.2025.101022 (PMC12803917; doi:10.1016/j.xops.2025.101022)
Supplement: Table S2 [file mmc3.pdf]

Supplementary Table 2. Slope of quality-of-life measures pre- and post-development of geographic atrophy, and difference between pre- and post-development slopes.

	**CGA**		**NCGA**		**Any GA**	
	98 eyes of 90 participants		260 eyes of 226 participants		358 eyes of 298 participants	
	Slope [95% CI]	*P* value	Slope [95% CI]	*P* value	Slope [95% CI]	*P* value
M2C pre	-0.03 [-0.12, 0.06]	0.50	-0.04 [-0.09, 0.01]	0.14	-0.02 [-0.06, 0.02]	0.35
M2C post	-0.09 [-0.21, 0.04]	0.20	-0.09 [-0.15, -0.03]	0.005	-0.08 [-0.13, -0.02]	0.006
M2C diff	-0.06 [-0.21, 0.10]	0.48	-0.05 [-0.13, 0.03]	0.19	-0.06 [-0.12, 0.01]	0.10
M2VF pre	-0.04 [-0.13, 0.06]	0.45	-0.02 [-0.08, 0.04]	0.52	-0.01 [-0.06, 0.04]	0.65
M2VF post	-0.12 [-0.26, 0.02]	0.10	-0.12 [-0.19, -0.04]	0.002	-0.10 [-0.16, -0.04]	0.002
M2VF diff	-0.08 [-0.25, 0.08]	0.33	-0.10 [-0.18, -0.01]	0.03	-0.09 [-0.16, -0.01]	0.02
M2SE pre	-0.02 [-0.17, 0.12]	0.74	-0.16 [-0.25, -0.07]	0.001	-0.10 [-0.17, -0.03]	0.007
M2SE post	-0.03 [-0.25, 0.20]	0.83	-0.05 [-0.15, 0.05]	0.35	-0.03 [-0.12, 0.06]	0.48
M2SE diff	0.00 [-0.30, 0.29]	0.99	0.11 [-0.03, 0.25]	0.12	0.07 [-0.05, 0.19]	0.27
Composite pre	-0.51 [-1.42, 0.40]	0.27	-0.57 [-1.03, -0.11]	0.02	-0.44 [-0.85, -0.03]	0.03
Composite post	-0.74 [-2.03, 0.55]	0.26	-1.35 [-1.94, -0.76]	<.001	-1.12 [-1.65, -0.59]	<.001
Composite diff	-0.23 [-1.69, 1.22]	0.75	-0.78 [-1.47, -0.08]	0.03	-0.68 [-1.29, -0.07]	0.03

Abbreviations: CI, confidence interval; M2C, Rasch-calibrated overall score; M2VF, subscale score describing visual function; M2SE, subscale describing socioemotional function; NEI VFQ-25, National Eye Institute 25-item Visual Function Questionnaire.
