# Supplementary material for: Changes in Vision-Related Quality of Life before and after Geographic Atrophy Development in Age-Related Eye Disease Study Participants
Source: Ophthalmol Sci. 2025 Nov 25;6(2):101022. doi: 10.1016/j.xops.2025.101022 (PMC12803917; doi:10.1016/j.xops.2025.101022)
Supplement: Table S3 [file mmc4.pdf]

Supplementary Table 3. Mediation analysis for the association between GA area and VRQOL measures among all patients developing central or noncentral GA.

	Model 1: Total effect of GA area (adjusted for age, time, and fellow-eye GA status)		**Model 2: Direct effect of GA area** (adjusted for age, time, and fellow-eye GA status, plus VA)	
VRQOL measure	Estimate [95% CI]	*P* value	Estimate [95% CI]	*P* value
M2C	-0.06 [-0.08, -0.03]	<0.001	-0.04 [-0.07, -0.01]	0.006
M2VF	-0.07 [-0.10, -0.04]	<0.001	-0.05 [-0.07, -0.02]	0.003
M2SE	-0.05 [-0.09, -0.01]	0.02	-0.02 [-0.06, 0.02]	0.27
Composite	-0.60 [-0.86, -0.33]	<0.001	-0.46 [-0.73, -0.20]	<0.001

Abbreviations: CI, confidence interval; GA, geographic atrophy; M2C, Rasch-calibrated overall score; M2VF, subscale score describing visual function; M2SE, subscale describing socioemotional function; NEI VFQ-25, National Eye Institute 25-item Visual Function Questionnaire.
