# Supplementary material for: Changes in Vision-Related Quality of Life before and after Geographic Atrophy Development in Age-Related Eye Disease Study Participants
Source: Ophthalmol Sci. 2025 Nov 25;6(2):101022. doi: 10.1016/j.xops.2025.101022 (PMC12803917; doi:10.1016/j.xops.2025.101022)
Supplement: Table S4 [file mmc5.docx]

**Supplementary Table 5.** Mediation analysis for the association between GA area and VRQOL measures among all patients developing noncentral GA.

|  | Model 1: Total effect of GA area  (adjusted for age, time, and fellow-eye GA status) | | **Model 2: Direct effect of GA area**  (adjusted for age, time, and fellow-eye GA status, plus VA) | |
| --- | --- | --- | --- | --- |
| VRQOL measure | Estimate  [95% CI] | *P* value | Estimate  [95% CI] | *P* value |
| M2C | -0.05 [-0.09, -0.02] | 0.003 | -0.05 [-0.08, -0.01] | 0.012 |
| M2VF | -0.06 [-0.10, -0.02] | 0.003 | -0.05 [-0.08, -0.01] | 0.013 |
| M2SE | -0.05 [-0.10, -0.01] | 0.046 | -0.03 [-0.08, 0.01] | 0.16 |
| Composite | -0.56 [-0.87, -0.24] | <0.001 | -0.52 [-0.82, -0.21] | 0.001 |

Abbreviations: CI, confidence interval; GA, geographic atrophy; M2C, Rasch-calibrated overall score; M2VF, subscale score describing visual function; M2SE, subscale describing socioemotional function; NEI VFQ-25, National Eye Institute 25-item Visual Function Questionnaire
